# New Jersey State Dental Society—Resolutions of Respect to Dr. F. A. Levy

**Published:** 1893-06

**Authors:** 


### NEW JERSEY STATE DENTAL SOCIETY—RESOLUTIONS OF RESPECT TO DR. F. A. LEVY.

FREDERICK A. LEVY, D.D.S., born at Richmond, Va.; died at Orange, N. J., March 20, 1893.

At a special meeting of the New Jersey State Dental Society the following memorial and resolutions on the death of Dr. Frederick Arthur Levy were adopted.

WHEREAS, In the death of our esteemed associate, Dr. Frederick A. Levy, the New Jersey State Dental Society has lost one of its most worthy and honored members and one full of generous impulses. He possessed a kindly professional feeling, and was foremost with those who labored to advance the profession by filling its ranks with honorable, capable men. He was a devoted professional brother, sociable, kind, and charitable to an eminent degree. Therefore be it

*Resolved*, That we bow to the will of Almighty God, and yet desire to emphasize our grief in the death of our professional brother, and to bear testimony to his abilities and self-sacrificing spirit in the advancement of his profession; also,

*Resolved*, That a copy of these resolutions be spread upon the minutes of our Society, and that a copy be sent to the dental journals for publication.

(Signed) W. F. HOLBROOK,
R. M. SANGER,
J. ALLEN OSMUN,
*Committee.*

